# Pacing in proximity to scar during cardiac resynchronization therapy increases local dispersion of repolarization and susceptibility to ventricular arrhythmogenesis

**DOI:** 10.1016/j.hrthm.2019.03.027

**Published:** 2019-10

**Authors:** Caroline Mendonca Costa, Aurel Neic, Eric Kerfoot, Bradley Porter, Benjamin Sieniewicz, Justin Gould, Baldeep Sidhu, Zhong Chen, Gernot Plank, Christopher A. Rinaldi, Martin J. Bishop, Steven A. Niederer

**Affiliations:** ∗ King’s College London, London, United Kingdom; † Medical University of Graz, Graz, Austria; ‡ Guy’s and St Thomas’ Hospital, London, United Kingdom

**Keywords:** Cardiac resynchronization therapy, Infarct scar, Patient-specific modeling, Ventricular tachycardia

## Abstract

**Background:**

Cardiac resynchronization therapy (CRT) increases the risk of ventricular tachycardia (VT) in patients with ischemic cardiomyopathy (ICM) when the left ventricular (LV) epicardial lead is implanted in proximity to scar.

**Objective:**

The purpose of this study was to determine the mechanisms underpinning this risk by investigating the effects of pacing on local electrophysiology (EP) in relation to scar that provides a substrate for VT in ICM patients undergoing CRT.

**Methods:**

Imaging data from ICM patients (n = 24) undergoing CRT were used to create patient-specific LV anatomic computational models including scar morphology. Simulations of LV epicardial pacing at 0.2–4.5 cm from the scar were performed using EP models of chronic infarct and heart failure (HF). Dispersion of repolarization and the vulnerable window were computed as surrogates for VT risk.

**Results:**

Simulations predict that pacing in proximity to scar (0.2 cm) compared to more distant pacing to a scar (4.5 cm) significantly (*P* <.01) increased dispersion of repolarization in the vicinity of the scar and widened (*P* <.01) the vulnerable window, increasing the likelihood of unidirectional block. Moreover, slow conduction during HF further increased dispersion (∼194%). Analysis of variance and *post hoc* tests show significantly (*P* <.01) reduced repolarization dispersion when pacing ≥3.5 cm from the scar compared to pacing at 0.2 cm.

**Conclusion:**

Increased dispersion of repolarization in the vicinity of the scar and widening of the vulnerable window when pacing in proximity to scar provides a mechanistic explanation for VT induction in ICM-CRT with lead placement proximal to scar. Pacing 3.5 cm or more from scar may avoid increasing VT risk in ICM-CRT patients.

## Introduction

Cardiac resynchronization therapy (CRT) is an effective treatment of heart failure (HF). However, CRT-induced ventricular tachycardia (VT) has been reported in patients with ischemic cardiomyopathy (ICM).^1^, ^2^, ^3^, ^4^, ^5^

The likelihood of unidirectional block leading to reentry in ICM-CRT patients is elevated due to the presence of scar. Dense fibrotic scar core and a border zone (BZ) of remodeled myocardium^6^ leads to increased dispersion of repolarization and widening of the vulnerable window for unidirectional block, which facilitates reentry. Dispersion of repolarization is further increased by electrophysiological (EP) changes commonly found in HF, including slower^7^ or faster^8^ conduction and prolongation of action potential duration (APD).^9^ Pacing from a left ventricular (LV) lead in close proximity to a scar may alter the activation and repolarization pattern around the scar, increasing the risk of reentry in these patients despite the potential improvement in cardiac function due to CRT.^4^

The effect of LV pacing in proximity to scar and subsequent increase in VT risk is unclear, as detailed mapping and pacing at multiple specific locations within a single patient is inherently challenging. We therefore created patient-specific computational models of LV anatomy, scar, and BZ to investigate the role of pacing location relative to scar on VT risk. We ran EP simulations and computed dispersion of repolarization and the vulnerable window as surrogates for VT risk.

## Methods

### Patient cohort

Late gadolinium enhancement magnetic resonance imaging (LGE-MRI) data from 24 ICM-CRT patients were available for this study. After institutional research ethics committee approval was obtained,^10^ patients underwent LGE-MRI assessment before device implantation and at least 6 months after previous myocardial infarction.^10^ A series of short-axis slices was acquired with in-plane resolution of 0.6 × 0.6–1.37 × 1.37 mm^2^ and slice thickness of 8–20 mm. Demographic characteristics are listed in Table 1.

**Table 1:** Demographic characteristics

Characteristic	Value
Age (y)	69.1 ± 9.5
Sex (male)	20 (83)
LBBB	11 (46)
QRS duration (ms)	133.6 ± 25.7
LV ejection fraction (%)	30.8 ± 12.7

Values are given as mean ± SD or n (%).

LBBB = left bundle branch block; LV = left ventricle.

### Construction of patient-specific models

The pipeline for construction of the patient-specific models is described in the following sections and the resulting models are shown in Supplementary Figure S1.

#### Image segmentation and processing

LV endocardium and epicardium contours were manually drawn in each short-axis slice using the image segmentation software Eidolon.^11^ Scar and BZ were segmented as the regions with signal intensity above 3 and 2 SD from the mean signal intensity within healthy myocardium, respectively, as described previously.^10^ The 2-dimensional scar and BZ segmentations were reconstructed in 3 dimensions using a statistical shape reconstruction method.^12^ Due to the low in-plane resolution of the images and to artifacts in the right ventricle (RV), it was not possible to segment the RV wall in this cohort.

#### Model generation

A finite element tetrahedral mesh was generated by interpolating the LV endocardium and epicardium contours.^11^ This mesh was then refined to generate a fine (mean edge length 0.35 mm) and a coarse mesh (mean edge length 0.8 mm) using the C-GAL library. The 3-dimensional reconstructed scar and BZ segmentations were mapped onto the tetrahedral mesh and used to label mesh elements as scar, BZ, and healthy tissue. Myofiber orientations were assigned to each anatomic model using a rule-based method.^13^ Although the models do not include the RV, its absence should not substantially affect our results because the RV lead of a CRT device is implanted against the septum.

### EP models and parameters

Activation and repolarization sequences were simulated in the LV models using the reaction-eikonal model, which allows simulating EP models using coarse spatial resolutions, thus substantially reducing computational cost.^14^ However, this model is not suitable for conduction block simulations, as activation is triggered at a prescribed time given by the solution of the eikonal equation.^14^ Thus, the cardiac monodomain model was used instead in simulations of conduction block. Both models were coupled to the ten Tusscher^15^ model of human ventricular action potential (AP). Simulations were performed using the Cardiac Arrhythmia Research Package (CARP).^14^

Because no personalized EP data were available for the patients, we assigned EP parameters based on the literature. Conduction velocities (CVs) were prescribed to the model by tuning tissue conductivities using an automatic approach.^16^ Transversely isotropic CV of 0.67 and 0.3 m/s in the longitudinal and transverse directions, respectively, were prescribed to healthy tissue. The BZ was prescribed isotropic CV of 0.15 m/s corresponding to 50% of the transverse CV in healthy tissue, according to experimentally measured values in the canine epicardial BZ of chronic infarcts.^6^ The scar core was modeled as unexcitable nonconducting tissue, assuming it consists of predominantly collagenous nonconducting material. Unless stated otherwise, no transmural or apicobasal variation of ionic currents was considered.

APD prolongation^17^ and conduction slowing^17^, ^18^ have been reported in HF patients. We investigated the impact of EP changes commonly found in HF by prescribing CVs 20% slower or faster than healthy/BZ tissue, as reported in rabbit^8^ and canine^7^ HF models, respectively. In addition, an HF ventricular AP model was implemented by altering the density of ionic currents in the ten Tusscher model, leading to prolonged APD, as previously described.^9^

Our EP models were based on chronic infarct^6^ and HF^19^ characteristics reported in the literature. However, the EP characteristics of individual patients vary and thus are likely to affect simulation results. Therefore, we evaluated the impact of our EP modeling choices on dispersion of repolarization. Specifically, we evaluated the impact of a slow conducting BZ, apicobasal and transmural APD heterogeneity, different AP models, and propagation models (Supplementary Methods and Results).

### Pacing locations and protocol for simulations of dispersion of repolarization

**Figure 1:**
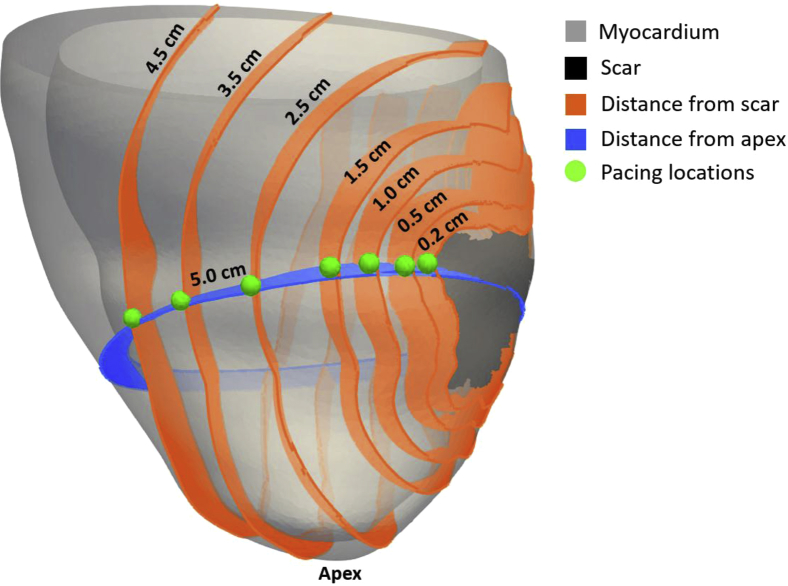
Pacing locations *(green)* relative to scar *(black). Orange* indicates distances from scar. *Blue plane* indicates the mid-scar plane located 5 cm from the apex.

Pacing locations at 0.2, 0.5, 1.0, 1.5, 2.5, 3.5, and 4.5 cm from the scar surface were chosen for each model at the mid-scar plane relative to the apex (Figure 1). These locations are in agreement with typical lead locations in CRT patients.^20^ Details on the distances computation are described in the Supplementary Methods and Results.

Activation was initiated at each pacing location using a point stimulus (∼1-mm circumference), and propagation was simulated for 1 second.

### Computing dispersion of repolarization

Repolarization times were computed as the time when the AP reached a threshold of –70 mV after activation. Local repolarization gradients were computed as the magnitude of the repolarization gradient vector at each mesh node.

We used the volume of tissue with high repolarization gradients (HRGs) as a metric of dispersion of repolarization and a surrogate for VT risk, assuming that the larger the tissue with HRG, the more likely unidirectional block is to occur in a given heart. A minimum local repolarization gradient of ∼3 ms/mm^21^ is required for the occurrence of unidirectional block. Thus, the volume of tissue with repolarization gradients above a threshold of 3 ms/mm was computed.

Arrhythmias in ICM patients typically originate within scar or BZ. Thus, we computed the volume of HRG within 0.5, 1.0, and 2.0 cm around the scar, and within the whole LV.

### Computing the vulnerable window

**Figure 2:**
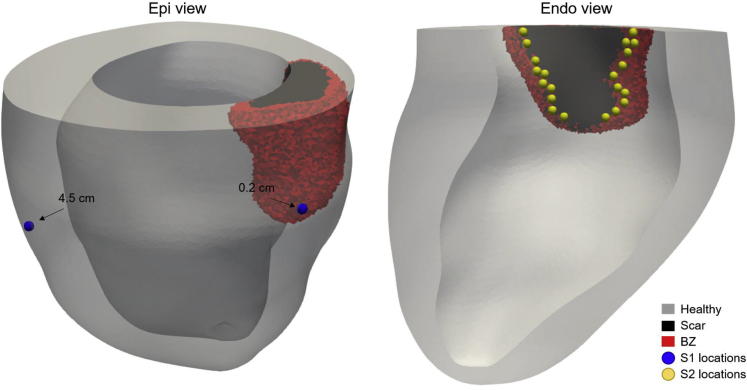
Location of S1 and S2 stimuli. **Left:** S1 stimuli locations at the epicardial surface 0.2 and 4.5 cm from the scar *(blue spheres).***Right:** Twenty-one S2 pacing locations *(yellow spheres)* selected on the endocardial surface within the border zone (BZ).

To investigate the role of pacing location relative to scar on the vulnerable window, we simulated premature beats after epicardial LV pacing at different locations. We used an S1-S2 protocol mimicking the occurrence of ectopic foci within the endocardial BZ, as observed in ICM. The S1 stimuli were delivered at the epicardial LV lead location 0.2 and 4.5 cm from the scar, and S2 pacing locations were chosen at the endocardial BZ ∼0.2 cm apart (Figure 2). Single premature S2 stimuli were applied at varying coupling intervals (CIs) after the last S1 beat.

The vulnerable window was computed at each S2 pacing location as the difference between the CI where a stimulus propagated and the smallest CI where unidirectional block occurred (Supplementary Methods and Results).

This part of the study was performed for only one of the patient-specific models due to the high computational cost of computing the vulnerable window. We chose a model with enough BZ at the endocardium to allow ∼20 S2 pacing locations spaced ∼0.2 cm apart.

### Statistical analysis

Balanced 1-way analysis of variance with Tukey-Kramer *post hoc* tests were used to compare the volume of HRG (within 0.5, 1, and 2 cm from scar and the whole LV) between the 7 pacing locations. Two sample paired Student *t* tests were used to compare the volume of HRG within 1 cm from the scar obtained with the HF models and to compare the vulnerable window when pacing 0.2 and 4.5 cm from the scar. Quantitative results are shown as boxplots. More details are described in the Supplementary Methods and Results.

## Results

### Effect of epicardial pacing on local dispersion of repolarization

**Figure 3:**
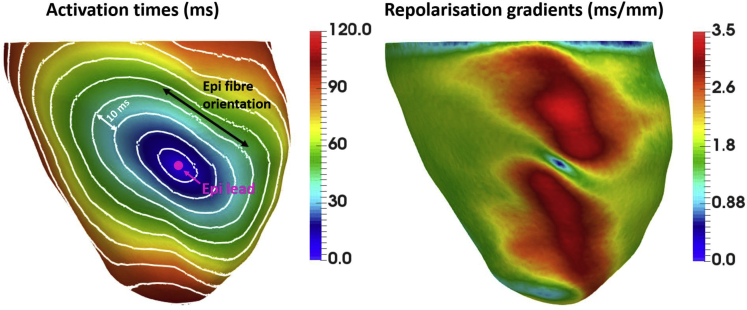
Activation times (ms) and repolarization gradients (ms/mm) after a point stimulus on the left ventricular epicardial surface of one of the models in our cohort, where the scar was modeled as healthy tissue. **Left:** Isochrones are 10 ms apart. Location of the epicardial lead is indicated by *pink circle.* Fiber orientation on the epicardial surface is indicated by the *black arrow.***Right:** Spatial distribution of local repolarization gradients corresponding to the activation sequence shown on the left. Large repolarization gradients *(red)* spread away from the pacing site in the direction transverse to fibers.

We investigated the effect of LV epicardial pacing on local repolarization gradients by simulating electrical propagation from an LV epicardial lead. This was done for one of the models in our cohort, where the scar was removed by modeling it as healthy tissue, thus isolating the effect of pacing. As shown in Figure 3, slower conduction transverse to the fiber orientation (left) leads to delayed repolarization in this direction, which in turn leads to large repolarization gradients (right). The local repolarization gradients are close to 0 ms/mm at the pacing site, as the tissue underneath the stimulus site activates nearly instantly, but increase to 1.8+ and 3.5+ ms/mm longitudinal and transverse to fibers, respectively.

### Effect of pacing location relative to scar on dispersion of repolarization

**Figure 4:**
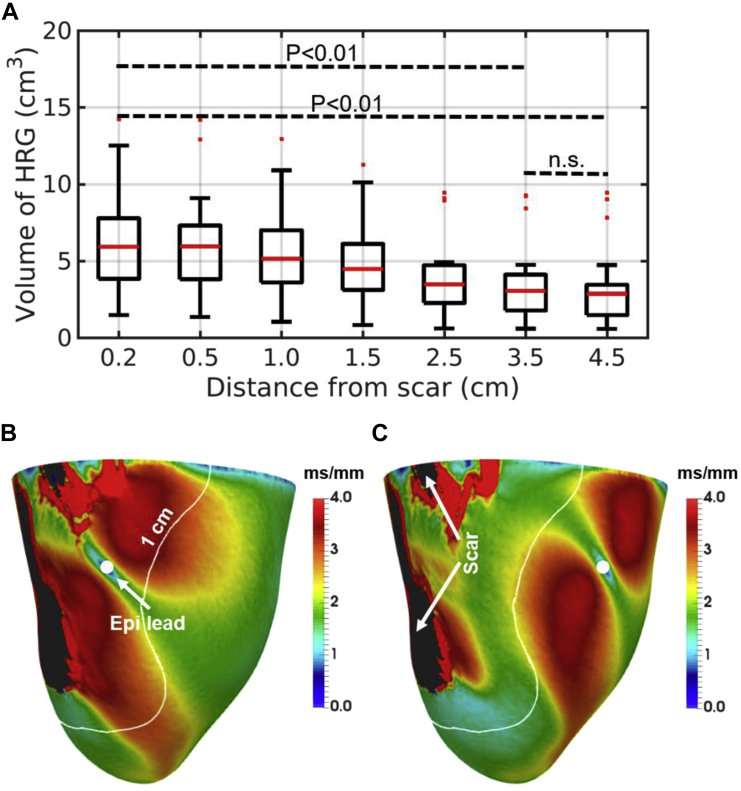
**A:** Volumes of high repolarization gradients (HRGs) within 1 cm around the scar relative to pacing distance from scar. *P* values are displayed. n.s. = nonsignificant. **B, C:** Example of repolarization gradients within the left ventricular (LV) epicardial surface for one of the models when pacing 0.2 cm **(B)** and 2.5 cm **(C)** from the scar. *White curves* indicate the region 1 cm around the scar. The core of the scar is shown in *black. Filled white circles* indicate the location of the LV epicardial lead.

We simulated repolarization sequences and computed the volume of HRG using 24 patient-specific models to investigate the role of LV epicardial pacing location relative to scar on dispersion of repolarization. Figure 4A shows the volumes of HRG within 1 cm around the scar for each pacing location. The plots show a clear trend toward decreased volumes of HRG within 1 cm around the scar when pacing away from the scar.

The effect of pacing in proximity to scar on the spatial distribution of repolarization gradients is shown in Figure 4 when pacing 0.2 (B) and 2.5 cm (C) from scar. Here, large repolarization gradients appear around the pacing site, as explained in the previous section, and in the vicinity of the scar, particularly within the BZ, where conduction is slower. Of note, the scale of 0 to 4 ms/mm for the repolarization gradients was chosen for visualization purposes only, and local gradients as high as 15 ms/mm were found in several cases.

The same trend, toward a smaller volume of HRG in the vicinity of the scar, is observed when considering regions 0.5, 1, and 2 cm around the scar (Supplementary Figure S2). Conversely, when considering the whole LV (Supplementary Figure S2), this trend disappears and even reverses when considering the pacing locations at 0.2 and 4.5 cm from the scar, where the total volume of HRG is larger when pacing 4.5 cm from the scar.

One-way analysis of variance shows statistically significant (*P* <.01) differences between pacing locations considering the regions within 1 and 2 cm from the scar. Tukey-Kramer *post hoc* tests for these regions show a significantly (*P* <.01) larger volume of HRG when pacing 0.2 cm from the scar compared to 3.5 and 4.5 cm (see Supplementary Methods and Results for details). Conversely, the difference in volume of HRG between 3.5 and 4.5 cm is not statistically significant.

### Effect of pacing location relative to scar on the vulnerable window

**Figure 5:**
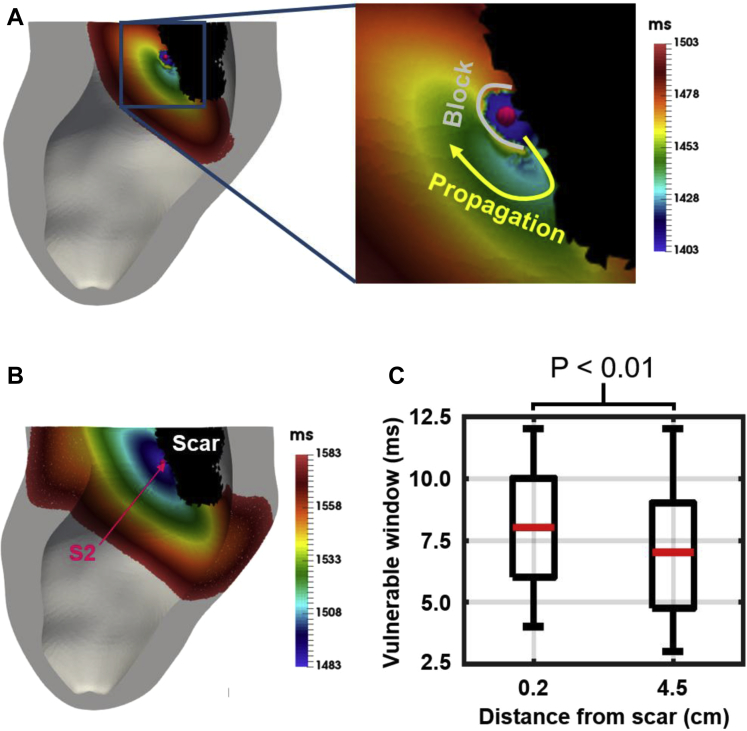
Example of unidirectional block **(A)** and normal propagation **(B)** after an S2 stimulus. **C:** Vulnerable window (ms) at each S2 pacing location when the left ventricular lead is located 0.2 and 4.5 cm from the scar. An increase in the vulnerable window is observed when pacing in proximity to scar compared to pacing away from it.

We used a patient-specific model to investigate the impact of pacing location on the vulnerable window. Figure 5A shows an example of unidirectional block after an S2 stimulus within the BZ at the endocardial surface. Here, the wavefront is blocked in the gray region, exits at a small region at the bottom of the stimulus, and travels around the region of conduction block. Figure 5B shows an example of normal propagation after an S2 stimulus for comparison. Figure 5C shows a boxplot of the vulnerable window computed at each premature S2 stimuli location when pacing (S1) at the epicardial surface 0.2 and 4.5 cm from the scar (see section on Computing the Vulnerable Window). A significant (*P* <.01) increase in the vulnerable window is observed when pacing 0.2 cm from the scar instead of 4.5 cm.

### Effect of EP changes found in HF

**Figure 6:**
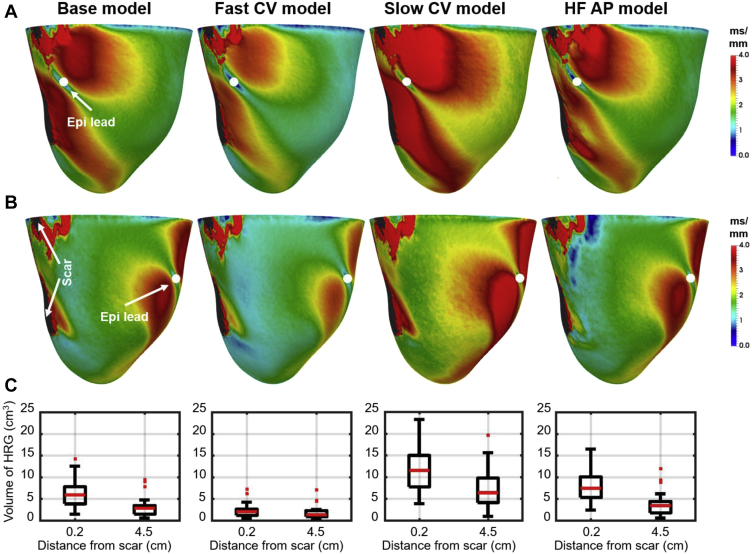
Effect of electrophysiological (EP) changes commonly found in heart failure (HF) on the volume of high repolarization gradients (HRG) within 1 cm around the scar when pacing 0.2 and 4.5 cm from the scar. From left to right: “Base model” refers to the model with normal conduction velocity (CV) within the left ventricle, slow CV within the border zone (BZ), and normal action potential (AP) morphology; “Fast CV model” and “Slow CV model” refer to models with 20% faster and slower CV within LV and BZ relative to values of the “Base model”, respectively; and “HF AP model” refers to the model with increased action potential duration . **A, B:** Examples of the spatial distribution of local repolarization gradients when pacing 0.2 and 4.5 cm from scar, respectively. **C:** Plots of the volume of HRG within 1 cm around the scar when pacing 0.2 and 4.5 cm from scar for each EP model.

We investigated the effect of EP changes commonly found in HF on the relationship between pacing location relative to scar and the volume of HRG using the HF models, with 20% faster CV (fast CV model), 20% slower CV (slow CV model), and with prolonged AP (HF AP model), as described in the section on EP Models and Parameters, and compared these with our “base model” with normal CV within the LV and slow CV in the BZ and with normal AP properties. Figure 6 shows the effect of each HF model on the volume of HRG within 1 cm from the scar when pacing 0.2 and 4.5 cm from the scar. Panels A and B show the spatial distribution of local repolarization gradients for 1 patient-specific model when pacing 0.2 and 4.5 cm from scar, respectively. Panel C show the volume of HRG for all patient-specific models and each HF model. The volume of HRG is significantly (*P* <.01) smaller when pacing 4.5 cm from scar than from 0.2 cm in all cases. The effect is most and least visible in the case of slower and faster conduction, respectively. APD prolongation has little effect compared with the base model.

## Discussion

Recent clinical studies provided empirical evidence that pacing in proximity to scar can cause VT in ICM-CRT patients.^4^ However, the underlying VT mechanisms remained unclear. Thus, we investigated the interaction between pacing electrode location relative to scar on VT risk in ICM-CRT patients. We used computational models of patient-specific anatomy and computed local repolarization gradients and the vulnerable window, as surrogates for VT risk, based on their known link to arrhythmogenesis. Our main finding is that pacing at the epicardial surface in proximity to scar increases the volume of HRG and widens the vulnerable window in the vicinity of the scar. This provides a plausible mechanistic explanation for increased VT risk in ICM-CRT patients. The risk of VT was further augmented by EP changes commonly found in HF.

### Mechanisms of increased VT risk when pacing in proximity to scar

When pacing at the epicardial surface via an LV lead (point stimulus), the propagating wavefront has a convex shape. A curved wavefront propagates more slowly than a planar wavefront, thus increasing dispersion of repolarization around the pacing site, particularly transverse to the fiber direction, where propagation is slower than parallel to it (Figure 4). When pacing in proximity to scar, the wavefront reaches the scar with larger curvature than when pacing away from it. This enhances dispersion of repolarization and increases the volume of HRG in the vicinity of the scar (Figure 4).

Increased dispersion of repolarization widens the vulnerable window for the occurrence of unidirectional block. Our study demonstrates that pacing in proximity to scar also widens the vulnerable window. Thus, there is both a larger volume of tissue and a longer time interval where unidirectional block may be induced when pacing in proximity to scar. Therefore, it is logical that pacing in proximity to scar will increase the likelihood of reentry in ICM-CRT patients, as observed clinically.^4^ Moreover, based on the trend of decreased volume of HRG in the vicinity of the scar when pacing away from it and on the *post hoc* test results, pacing ≥3.5 cm from the scar is likely to avoid increasing susceptibility to reentry.

It is worth noting that the morphology of the scar/BZ varies substantially in our cohort (Supplementary Figure S1) and is likely to influence the occurrence of reentry in an individual patient. Although we have computed the vulnerable window for a single patient, due to the large computational cost of the simulations, the volume of HRG was computed for all patients and thus account for variability of scar/BZ morphology.

### Impact of HF on VT risk

Slow CV reported in HF widens the vulnerable window, predisposing patients to reentry.^7^, ^17^, ^18^ This scenario may be particularly dangerous with increased heart size during HF, in which the wavelength to heart size ratio is reduced, thus facilitating reentry.^7^ Conversely, HF has also been associated with increased CV due to increased cell size.^8^ Although increased CV may be antiarrhythmogenic, as it increases the wavelength, it may facilitate reentry if the increase is heterogeneously distributed throughout the heart, increasing dispersion of repolarization.

Prolongation of APD^17^ due to reduced potassium currents^22^ is common in HF. Reduced potassium current reduces repolarization reserve, which facilitates the occurrence of early afterdepolarizations. At the tissue scale, local early afterdepolarizations can synchronize and propagate, leading to reentry, particularly in the scenario of decreased tissue coupling.^23^

In this study, we demonstrated that EP changes commonly found in HF affect the volume of HRG within 1 cm from scar, with slower conduction substantially increasing it (Figure 6). Conversely, the volume of HRG remains larger when pacing in proximity to scar. These results highlight that pacing in proximity to scar may increase VT risk regardless of the underlying EP properties but may be particularly harmful in the presence of slow conduction.

### Impact of model choice

In this study, we did not personalize EP, as invasive EP data, such as electroanatomic mapping, were not available. However, we investigated the impact of EP model choice and assumptions on our results using alternative cell models (ten Tusscher, O’Hara-Rudy, Grandi) (Supplementary Figure S3), the monodomain model (Supplementary Figure S4), and various model parameters that account for EP spatial heterogeneity (transmural and apicobasal APD heterogeneity and a slow conducting BZ) (Supplementary Figure S3). Our results (Supplementary Figures S3 and S4) show that, although the total volume of HRG varied with the presence of spatial EP heterogeneity and with different EP models, the relationship between pacing location and the volume of HRG is consistent and statistically significant across all models, with smaller volume when pacing away from the scar.

### Comparison with previous studies

Recent clinical studies have described CRT-induced arrhythmias,^1^, ^2^, ^3^, ^4^, ^5^ particularly in ICM patients^1^, ^2^, ^3^, ^5^ and when pacing near scar.^4^ However, the underlying EP changes induced by pacing near scar that give rise to VT remained unknown. To the best of our knowledge, this is the first study to provide a mechanistic explanation for increased VT risk in ICM-CRT patients when pacing in proximity to scar. Our results are in agreement with the experimental and clinical literature, which suggests that patients with scar and HF are intrinsically at higher risk for VT due to EP changes and that this is further enhanced by pacing in proximity to scar.^4^

### CRT in ICM patients

Current CRT guidelines recommend that pacing leads should be implanted away from scar to improve response to treatment, with reports of VT when pacing near scar^4^ further strengthening the argument against pacing in the proximity of scar. However, how far from a scar is safe to pace remained unknown. We showed that pacing away from a scar decreases the volume of HRG in its vicinity, particularly pacing ≥3.5 cm from the scar. This suggests that pacing 3.5 cm or more from a scar may avoid increasing VT risk in ICM-CRT patients.

Epicardial lead placement is limited by the anatomy of the coronary sinus, which makes it difficult to avoid pacing in/near scar. Moreover, pacing away from a scar may result in suboptimal resynchronization. Multisite and endocardial pacing may offer feasible alternatives to conventional CRT in ICM patients. Multisite pacing allows pacing from multiple locations, maximizing resynchronization, and modifying these over time should they prove arrhythmogenic.^3^ Endocardial pacing allows pacing anywhere within the endocardial surface, thus facilitating pacing within an optimal region while avoiding scar. In addition, measuring the stimulus to QRS interval may allow identifying optimal pacing sites while avoiding scar.^24^

Of note, the impact of pacing in proximity to the scar was independent of scar location within the LV. In 17 patients who had scar in the septum, pacing in proximity to the scar caused the same increase in volume of HRG as when the scar was located in the LV free wall. This would support also placing the RV lead away from scar if possible.

## Conclusion

LV epicardial pacing in proximity to scar increased the volume of tissue with HRG and widened the vulnerable window for unidirectional block. In addition, the presence of slow conduction, commonly found in HF, further increased the volume of HRG. Thus, our study provides a plausible mechanistic explanation for increased VT risk in ICM-CRT patients when pacing in proximity to scar. Our results also suggest that pacing ≥3.5 cm from the scar may avoid increasing VT risk in ICM-CRT patients.
