# Supplementary material for: Pacing in proximity to scar during cardiac resynchronization therapy increases local dispersion of repolarization and susceptibility to ventricular arrhythmogenesis
Source: Heart Rhythm. 2019 Oct;16(10):1475–83. doi: 10.1016/j.hrthm.2019.03.027 (PMC6774764; doi:10.1016/j.hrthm.2019.03.027)
Supplement: Supplementary Material [file mmc1.docx]

**Supplementary methods and results**

Pacing in proximity to scar during cardiac resynchronization therapy increases local dispersion of repolarization and increases susceptibility to ventricular arrhythmogenesis.

Caroline Mendonca Costa, Aurel Neic, Eric Kerfoot, Bradley Porter, Benjamin Sieniewicz, Justin Gould, Baldeep Sidhu, Zhong Chen, Gernot Plank, Christopher A. Rinaldi, Martin J. Bishop, Steven A. Niederer

1. **Patient-specific models**


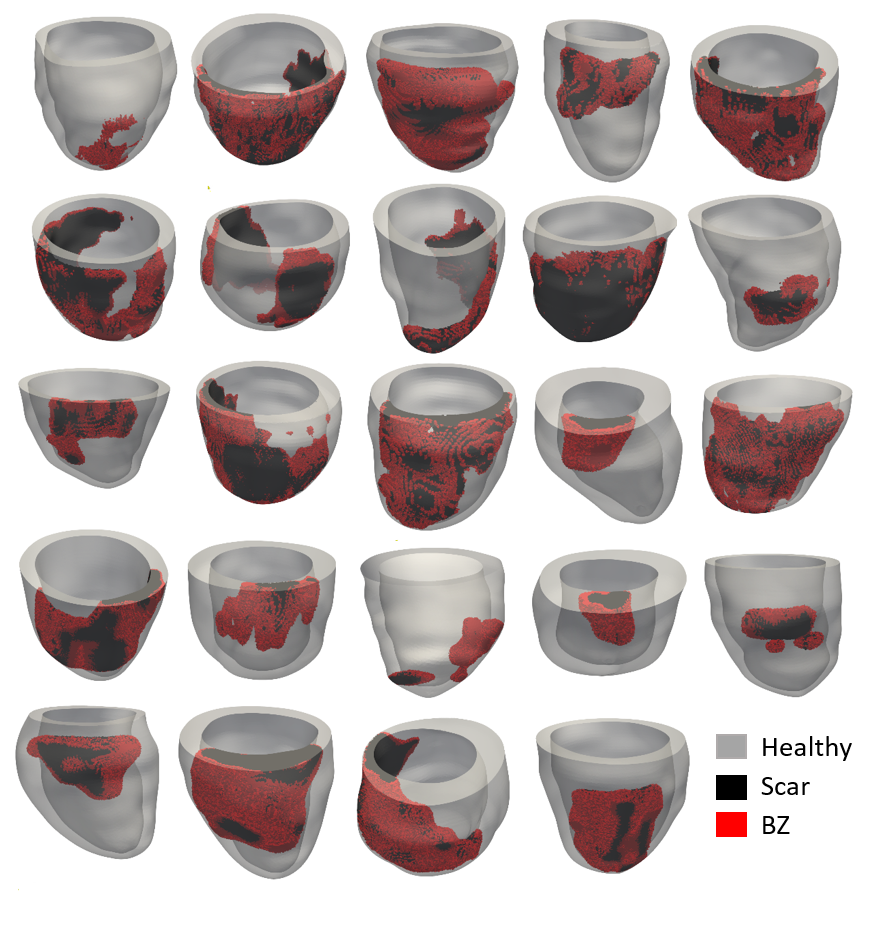


Figure S1: 24 personalized models of left ventricular anatomy, scar, and border zone (BZ). Healthy tissue is shown in gray, scar in black and BZ in red

1. **Computing distances from scar and apex**

Distances from scar were computed by simulating activation sequences based on the Eikonal equation. Activation was initiated at all vertices on the scar surface and the core of scar was set as non-conducting. The conduction velocity was set to 1 m/s, thus giving activation time equals distance. The distance from the apex was computed in the same way but activation was initiated at the apex instead of the scar surface and the scar was treated as normal instead of non-conducting tissue.

1. **Computing the vulnerable window**

The vulnerable window was computed at each S2 pacing location using an iterative approach, where simulations were performed in three steps. First, a “no capture” simulation was run, where an S2 stimulus was delivered 10 ms before the tissue repolarized and thus did not propagate. Second, a “propagation” simulation was run, where an S2 stimulus was delivered 100 ms after the repolarized and thus propagated normally. Finally, S2 stimuli were delivered at increasing CI (1 ms intervals) starting from the “no capture” CI. Propagation following each S2 stimuli was simulated for 100 ms. The activation times of each simulation were computed and converted to a binary map (activated or not activated). A Dice Similarity Coefficient (DSC) was used to compare the activation pattern of each S2 simulation and a stimulus was classified as either “no capture”, “uni-directional block”, or “propagation”. The vulnerable window was then computed as the difference between the CI for the first “propagation” stimulus and the CI of the first “uni-directional block” stimulus.

1. **Volume of high repolarization gradients for different regions around the scar**

We computed the volume of high repolarization gradients (HRG) 0.5, 1, and 2 cm around the scar and within the whole left ventricle (LV). In Figure S2, a clear trend towards a smaller volume of HRG when pacing away compared to pacing near the scar is observed when considering the regions up to 2 cm around the scar. The trend disappears and slightly reverses when considering the whole LV. This highlights that the observed trend is a localised phenomenon and thus cannot be captured by global metrics of dispersion of repolarization.

One-way ANOVA shows a statistically significant (P<0.01) difference in volume of HRG between pacing locations for the regions within 1 and 2 cm from the scar, but not for the region 0.5 cm from the scar or the whole LV. Post-hoc tests for region 1 and 2 cm from scar show a significant (P<0.01) difference between the pacing location 0.2 cm from the scar and the locations 3.5 and 4.5 cm from the scar, as highlighted in Figure S2. In addition, when considering a significance level of 0.05, the test shows a significant (P<0.05) difference between the pacing location 0.2 cm and the locations 2.5, 3.5 and 4.5 cm. Conversely, the volume of HRG between pacing locations 2.5, 3.5 and 4.5 cm from the scar are not significantly different (P>0.05). This suggests that pacing more than 3.5 cm from the scar may avoid increasing susceptibility to arrhythmia in ICM-CRT patients.


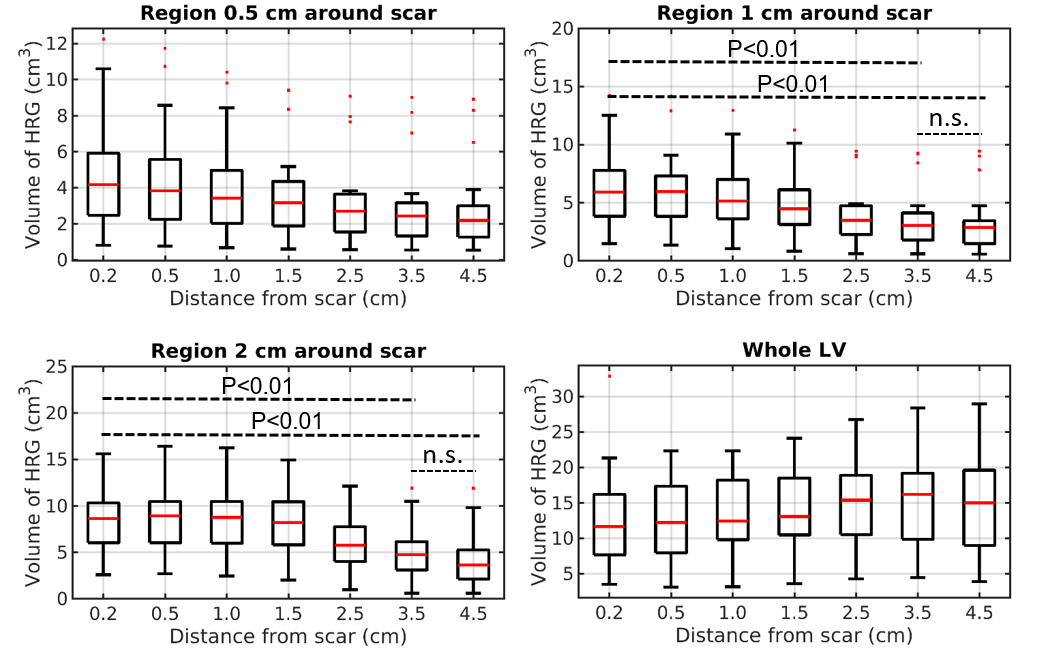


Figure S2: Volume of high repolarization gradients (HRG) within 0.5, 1, and 2 cm around the scar and within the whole left ventricle (LV) when pacing at different distances from the scar. n.s stands for non-significant.

1. **Alternative electrophysiological models**

Alternative models were also considered to evaluate the impact of modelling choices on the volume of HRG and its relationship with pacing location relative to scar. The impact of transmural and apico-basal action potential heterogeneity on the volume of HRG was evaluated by altering the action potential duration of the ten Tusscher model^1^ using a linear function, as done previously^2^. The impact of the BZ was evaluated by setting normal conduction velocity within the BZ region. Two other action potential models of human ventricular cells were tested, namely the O’Hara model^3^ and the Grandi model^4^.

Figure S3 shows that, although the specific volume of HRG varying for different models, its relationship with pacing location is preserved, with the volume of HRG smaller when pacing 4.5 cm from the scar compared with pacing 0.2 cm from it. Paired t-tests revealed that the difference in volume of HRG between the two pacing locations are statistically significant (P < 0.01) for all alternative models.


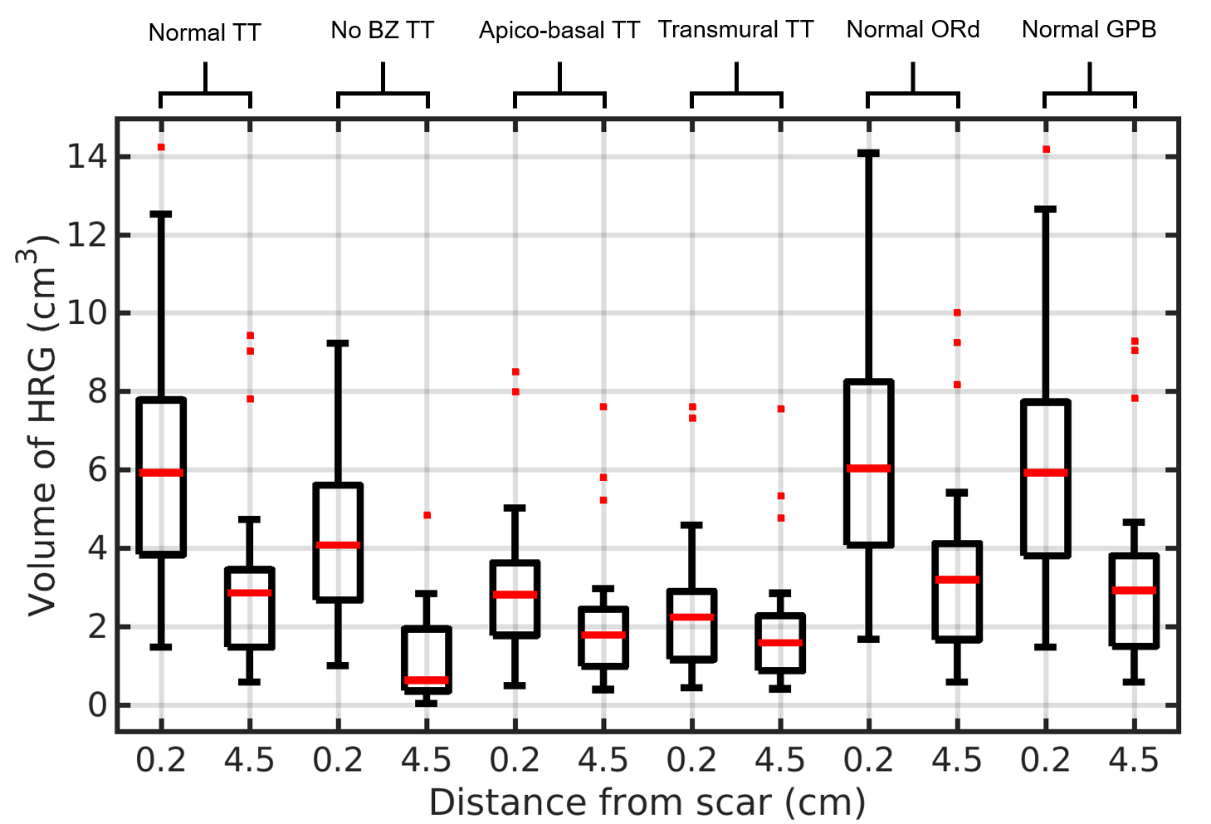


Figure S3: Impact of modelling choices on the volume of high repolarization gradients (HRG) within 1 cm around the scar and its relationship with pacing location

Monodomain simulations were also performed using a finer model (mean edge length of 0.35 mm). Three consecutive beats with a basic cycle length of 500 ms were simulated and repolarization times and local gradients computed, as previously described. Simulations were run when pacing 0.2 and 4.5 cm from the scar. The volumes of HRG (≥ 3ms/mm) were computed for each beat. Figure S4 shows the spatial distribution of local repolarization gradients and the computed volumes of HRG for three consecutive beats. Here, the volume of HRG is smaller when pacing 4.5 cm from the scar, as for the simulations with the Reaction-Eikonal model and all other alternative EP models. A slight decrease in the volumes is observed after the first beat, but the same trend remains.


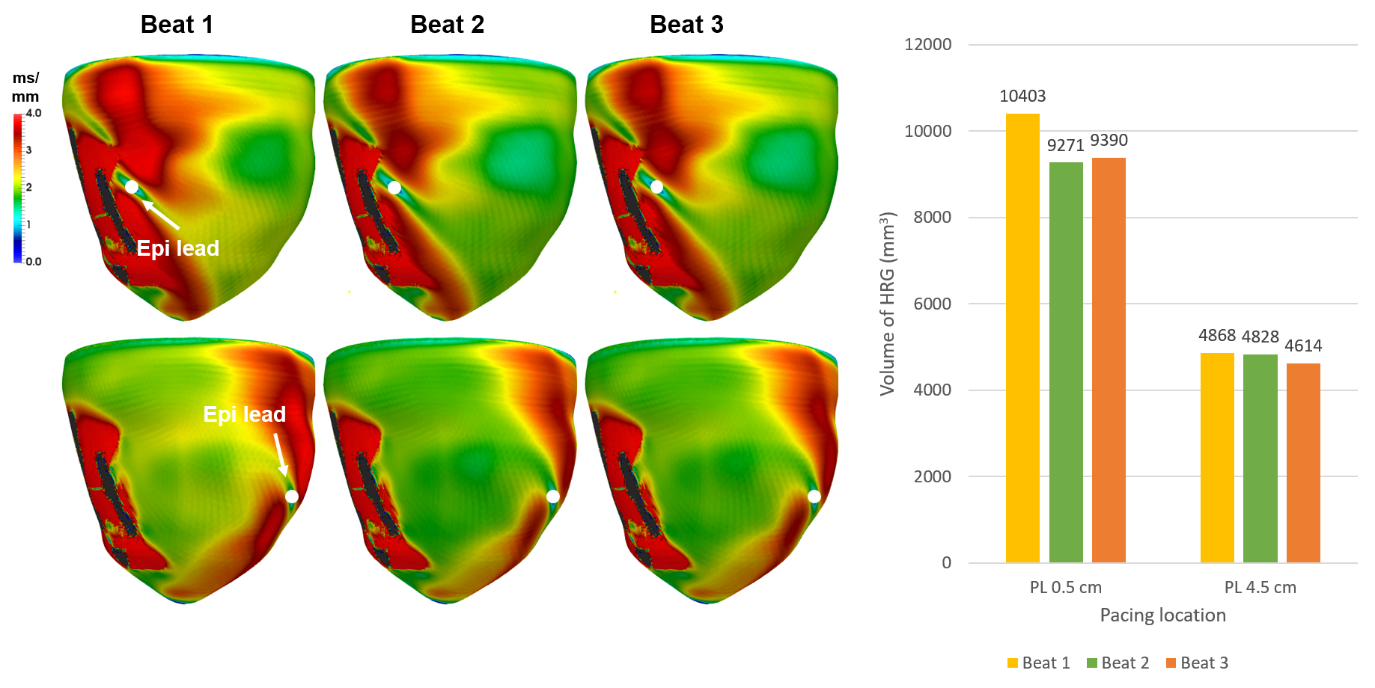


Figure S4: Spatial distribution of repolarization gradients (left) and volume of high repolarization gradients (HRG) within 1 cm around the scar using the monodomain model and following three consecutive beats.

1. **Details of the box plots**

Quantitative results are shown as boxplots, where the median is indicated as a red line within the box. The top and bottom edges of the box’s whiskers represent the 75 and 25 percentile, respectively. The whiskers extend to the most extreme points that are not considered outliers. The maximum whisker length, W, is 1.5. Points are draw as outliers if they are larger than Q3+W(Q3-Q1) or smaller than Q1-W(Q3-Q1), where Q1 and Q3 are the 25% and 75% percentiles, respectively. Outliers are plotted separately as red markers.

**References**

1. Ten Tusscher KHWJ, Panfilov A V, Tusscher T. Alternans and spiral breakup in a human ventricular tissue model. *Am J Physiol Hear Circ Physiol*. 2006;291:1088-1100. doi:10.1152/ajpheart.00109.2006.

2. Keller DUJ, Weiss DL, Dossel O, Seemann G. Influence of I Ks heterogeneities on the genesis of the T-wave: A computational evaluation. *IEEE Trans Biomed Eng*. 2012;59(2):311-322. doi:10.1109/TBME.2011.2168397.

3. O’Hara T, Virág L, Varró A, Rudy Y. Simulation of the undiseased human cardiac ventricular action potential: Model formulation and experimental validation. *PLoS Comput Biol*. 2011;7(5):1002061. doi:10.1371/journal.pcbi.1002061.

4. Grandi E, Pasqualini FS, Bers DM. A novel computational model of the human ventricular action potential and Ca transient. 2009. doi:10.1016/j.yjmcc.2009.09.019.
